# *Plasmodium vivax* HAP2/GCS1 gene exhibits limited genetic diversity among parasite isolates from the Greater Mekong Subregion

**DOI:** 10.1186/s13071-020-04050-0

**Published:** 2020-04-07

**Authors:** Danni Li, Chunyun Yu, Jian Guo, Yazhou Wang, Yan Zhao, Lin Wang, Myat Thu Soe, Hui Feng, Myat Phone Kyaw, Jetsumon Sattabongkot, Lubin Jiang, Liwang Cui, Xiaotong Zhu, Yaming Cao

**Affiliations:** 1 Department of Immunology, College of Basic Medical Science, China Medical University, Shenyang, 110122 Liaoning Province People’s Republic of China; 2 Department of Laboratory Medicine, Shanghai East Hospital, Tongji School of Medicine, Shanghai, People’s Republic of China; 3 Department of Environmental Health, School of Public Health, China Medical University, Shenyang, 110122 Liaoning Province People’s Republic of China; 4 Myanmar Health Network Organization, Yangon, Myanmar; 5 Mahidol Vivax Research Unit, Faculty of Tropical Medicine, Mahidol University, Bangkok, Thailand; 6 Unit of Human Parasite Molecular and Cell Biology, Key Laboratory of Molecular Virology and Immunology, Institut Pasteur of Shanghai, Chinese Academy of Sciences, Shanghai, People’s Republic of China; 7 Department of Internal Medicine, Morsani College of Medicine, University of South Florida, 3720 Spectrum Boulevard, Suite 304, Tampa, FL 33612 USA

**Keywords:** *Plasmodium vivax*, HAP2/GCS1, Transmission-blocking vaccine, Genetic diversity

## Abstract

**Background:**

Antigens expressed in sexual stages of the malaria parasites are targets of transmission-blocking vaccines (TBVs). HAP2/GCS1, a TBV candidate, is critical for fertilization in *Plasmodium*. Here, the genetic diversity of PvHAP2 was studied in *Plasmodium vivax* parasite populations from the Greater Mekong Subregion (GMS).

**Methods:**

*Plasmodium vivax* clinical isolates were collected in clinics from the China-Myanmar border region (135 samples), western Thailand (41 samples) and western Myanmar (51 samples). Near full-length *Pvhap2* (nucleotides 13–2574) was amplified and sequenced from these isolates. Molecular evolution studies were conducted to evaluate the genetic diversity, selection and population differentiation.

**Results:**

Sequencing of the *pvhap2* gene for a total of 227 samples from the three *P. vivax* populations revealed limited genetic diversity of this gene in the GMS (π = 0.00036 ± 0.00003), with the highest π value observed in Myanmar (0.00053 ± 0.00009). Y133S was the dominant mutation in the China-Myanmar border (99.26%), Myanmar (100%) and Thailand (95.12%). Results of all neutrality tests were negative for all the three populations, suggesting the possible action of purifying selection. Codon-based tests identified specific codons which are under purifying or positive selections. Wright’s fixation index showed low to moderate genetic differentiation of *P. vivax* populations in the GMS, with *F*_ST_ ranging from 0.04077 to 0.24833, whereas high levels of genetic differentiation were detected between the China-Myanmar border and Iran populations (*F*_ST_ = 0.60266), and between Thailand and Iran populations (*F*_ST_ = 0.44161). A total of 20 haplotypes were identified, with H2 being the abundant haplotype in China-Myanmar border, Myanmar and Thailand populations. Epitope mapping prediction of Pvhap2 antigen showed that high-score B-cell epitopes are located in the S307-G324, L429-P453 and V623-D637 regions. The E317K and D637N mutations located within S307-G324 and V623-D637 epitopes slightly reduced the predicted score for potential epitopes.

**Conclusions:**

The present study showed a very low level of genetic diversity of *pvhap2* gene among *P. vivax* populations in the Greater Mekong Subregion. The relative conservation of *pvhap2* supports further evaluation of a Pvhap2-based TBV.
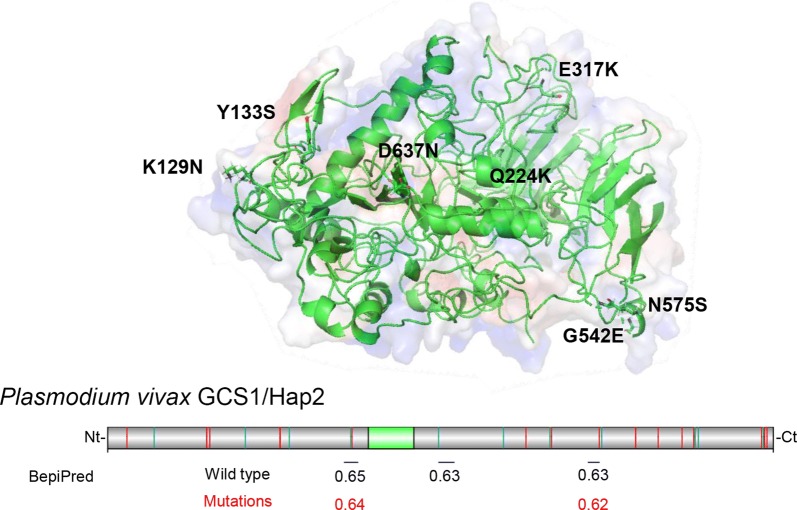

## Background

Malaria remains an important public health problem in the six countries of the Greater Mekong Subregion (GMS), including Cambodia, China’s Yunnan and Guangxi provinces, Lao PDR, Myanmar, Thailand and Vietnam [1]. Launching of the malaria elimination programme in the GMS has led to a steady decline of malaria burden in the GMS in recent years [2]. However, this ambitious plan encounters multiple challenges such as antimalarial multidrug resistance, cross-border population mobility and parasite introduction, and asymptomatic *Plasmodium* infections as the reservoir for malaria transmission [3–6]. In addition, *Plasmodium vivax* has become the predominant parasite in the GMS, and its proportion has been increasing [7]. Thus, new and integrated intervention strategies to reduce malaria transmission are needed. Among them, transmission-blocking vaccines (TBVs) are considered an integral component of the measures for malaria control and elimination [8–11].

Gametocytes developed in the human host are the obligative stage for the transmission to mosquitoes [12]. After a mosquito takes an infected blood meal, male and female gametocytes are activated in the mosquito midgut to develop into gametes. Fertilization between a microgamete and a macrogamete produces a zygote, which then transforms into a mobile ookinete. The ookinete traverses the mosquito midgut wall and starts sporogonic development in the mosquito. Although the molecular mechanisms of fertilization are poorly understood, this process is a promising target for the interruption of transmission [13]. Several proteins involved in the fertilization process have been identified, including P47, P48/45, P230 and HAP2 [14, 15]. HAP2/Generative Cell Specific 1 (GCS1), first identified in the plants *Arabidopsis thaliana* and *Lilium longflorum*, is a class II viral fusion protein with a cysteine-rich extracellular region [16–18]. HAP2/GCS1 is highly conserved in a wide range of species, such as the green alga *Chlamydomonas* and *Plasmodium* [19]. The N-terminus of this protein interacts with proteins expressed on the female gamete, whereas the C-terminus interacts with sperm plasma membrane during fertilization [20]. In *Plasmodium*, HAP2 protein is located specifically on the surface of activated male gametes [18, 19]. It is an essential component for gamete fusion, since *hap2* gene knockout in the rodent parasite *P. berghei* abolishes fertilization [19, 21]. Recent studies identified a conserved ‘cd loop’ segment within the HAP2/GCS1 protein, corresponding to amino acids (aa) 174–205 in PbHAP2 and aa 178–207 in *P. falciparum* HAP2, which is critical for membrane fusion during fertilization [16, 22, 23]. Antibodies raised against ‘cd loop’ segment of both PbHAP2 and PfHAP2 protein showed evident transmission reduction activity (TRA) in mosquito feeding assays [21, 24]. Meanwhile, like other surface-located vaccine candidate antigens, naturally acquired antibodies against HAP2 are detected in malaria exposed individuals [24]. Altogether, these results suggest that HAP2 is a promising TBV candidate.

Understanding the genetic diversity of a vaccine candidate in endemic areas is necessary for guiding the development of an effective malaria vaccine. Genetic polymorphism in vaccine candidates for asexual stages of malaria parasites has been a major limiting factor for the development of malaria vaccines [25]. Thus, the genetic diversity, natural selection, population differentiation and haplotype prevalence of Pvhap2 in *P. vivax* populations from the GMS were assessed in this study. These analyses revealed limited genetic diversity of *Pvhap2* gene as well as little differentiation of parasite populations in the GMS compared with the global sequences of a gamete fertilization essential gene, so as to provide useful information for transmission blocking vaccine development.

## Methods

### *Plasmodium vivax* clinical isolates

A total of 135, 51 and 41 clinical parasite isolates were collected from patients with acute *P. vivax* malaria attending clinics located in the China-Myanmar border area (Laiza Township, Kachin State, Myanmar and Nabang Township, Yunnan Province, China) in 2016, western Myanmar (Paletwa Township, Chin State) in 2018, and western Thailand (Tha Song Yang District, Tak Province) in 2011, respectively (Additional file 1: Figure S1). The distances among three sampling sites are similar (1275–1450 km). *Plasmodium vivax* infections were diagnosed by microscopical examination of Giemsa-stained thin and thick blood smears. Filter paper blood spots from finger-prick were prepared and stored individually in sealed plastic bags. Written informed consent was obtained from all participants and guardians of children.

### Genomic DNA extraction, PCR and sequencing of the *pvhap2* gene

Genomic DNA (gDNA) was extracted from dried filter blood spots by using the QIAamp DNA mini kit (Qiagen, Hilden, Germany) and stored at − 20 °C until use. Primers for *pvhap2* gene amplification were designed using the *pvhap2* sequence (PlasmoDB ID: PVX_094925), located on chromosome 8 at nucleotides 651,355 to 654,032 of the *P. vivax* Salvador-I (Sal I) reference strain (Additional file 2: Table S1). The *pvhap2* sequence corresponding to nucleotides 13–2574 (aa 5–858) was amplified by PCR using the primer pair pvsHap2-1F and pvsHap2-1R for the primary PCR, and the primer pair pvsHap2-nest-F and pvsHap2-nest-R for the nested PCR. Primary and nested PCR was conducted in a reaction volume of 20 μl including 1× KOD-Plus-Neo buffer (Toyobo, Osaka, Japan), 200 μM dNTPs (Toyobo), 1 mM MgSO_4_ (Toyobo), 10 µM of each primer, 1 μl of gDNA or primary PCR product (1:1000 dilution), and 0.4 units of KOD-Plus-Neo DNA polymerase (Toyobo). The cycling conditions for both primary and nested PCR were as follows: one step at 95 °C for 5 min; 39 cycles at 95 °C for 30 s, 55 °C for 15 s, and 68 °C for 1 min; and an extension step at 68 °C for 5 min. The PCR products were separated in 1.5% agarose gel in the presence of SYBR^TM^ Safe DNA (Thermo Fisher Scientific, Waltham, USA) and visualized under UV light. PCR fragments were purified with QIAquick PCR Purification kit (Qiagen) according to the manufacturer’s protocol and sequenced in both directions using primers listed in Additional file 2: Table S1 on a 3730XL DNA analyzer (Applied Biosystems, Waltham, USA). To verify the sequencing accuracy, purified PCR products from two independent amplifications of each isolate were sequenced in both DNA strands. Sequencing results of samples with double peaks at one single site were considered as mixed infection and excluded from further analysis. All nucleotide singletons (substitutions appearing only once among the sequences) were re-sequenced from new PCR products for verification. The nucleotide sequences were deposited in the GenBank database under the accession numbers MT087312-MT087446 (China-Myanmar border), MT087488-MT087538 (Myanmar) and MT087447-MT087487 (Thailand). In addition, 52 *pvhap2* sequences from Iranian *P. vivax* isolates (GenBank: KM594259-KM594288 and KU743361-KU743382) were included for comparison [26].

### Genetic diversity and natural selection

*Pvhap2* sequences were aligned using Clustal W program in MEGA X [27]. The number of segregation sites (*S*), total number of mutations (*η*), nucleotide diversity (π), the average number of nucleotide differences (*k*), number of haplotypes (H), haplotype diversity (Hd) and the corresponding standard deviation were computed using DnaSP v6.10.01 [28]. Additionally, nucleotide diversity was also estimated on a sliding window of 90 bases with a step size of 3 bp implemented in DnaSP v6.10.01 [28]. Natural selection was determined by calculating the *d*_N_*-d*_S_ difference using the Nei & Gojobori’s method with the Jukes & Cantor correction as implemented in MEGA X, and significance was determined by a *Z*-test [27]. A positive value of *d*_N_-*d*_S_ shows positive natural selection, whereas a negative value indicates purifying selection. The Tajima’s *D* test [29], Fu & Liʼs D^*^ and F^*^ tests [30] were performed using DnaSP v6.10.01 to determine departure from neutrality [28]. McDonald-Kreitman (MK) test [31] was performed using the *Plasmodium cynomolgi hap2* gene (PlasmoDB ID: PcyM_0814900) as an outgroup [28]. Meanwhile, to detect selection acting on specific amino acids of the protein, codon-based test was performed by using HyPhy package implemented in the Data Monkey Web Server [32]. Fisher’s exact test was applied to test for significant non-randomness (*P* < 0.05), and the skew from randomness was calculated as the neutrality index.

### Genetic differentiation, haplotype network and phylogenetic analysis

The genetic differences between populations was investigated by estimating the rate of fixation (*F*_ST_) implemented in ARLEQUIN v3.5.2.2 software [33]. Interpretation of *F*_ST_ values is defined as no differentiation (0), low genetic differentiation (≤ 0.15), moderate genetic differentiation (0.15–0.25) and high differentiation (≥ 0.25) [34]. *P*-value < 0.05 was considered as indicating a significant difference. The median-joining method in NETWORK v5.0.0.3 [35] was used to establish genealogical relationship of the Pvhap2 haplotypes among the parasite populations. A phylogenetic tree was constructed using the neighbor joining method in MEGA X software with a bootstrap of 1000 pseudo-replicates [27].

### Prediction of linear B cell and T cell epitopes

The potential B cell epitopes of Pvhap2 were predicted by using the ABCpred server [36] and BepiPred (http://www.cbs.dtu.dk/services/BepiPred/). For ABCpred server, a threshold of 0.7 was used to predict a peptide length of 16 residues. The program BepiPred was run with an epitope threshold of 0.6, and the epitope score represents the average of the scores of at least eight consecutive amino acids above the cut-off. The overlapped predicted regions from ABCpred and BepiPred, as well as mutation sites were marked on the schematic structure of Pvhap2 protein by using DOG 2.0 software [37, 38]. The 3D structure of the full-length Pvhap2 was predicted using the Phyre^2^ algorithm [39]. The electrostatic potential of Pvhap2 3D structure was calculated with the Adaptive Poisson-Boltzmann Solver software integrated with Pymol2.3 program [40]. The amino acid sequence of Pvhap2 in the Sal I strain (PlasmoDB ID: PVX_094925) was used.

## Results

### Polymorphism in the *pvhap2* gene

We sequenced the almost full-length *pvhap2* gene (nt 13–2574 bp) in 227 clinical *P. vivax* samples collected from three regions of the GMS. From the alignment with the reference Sal I strain, 28 single nucleotide polymorphisms (SNPs) were identified. Among them, 16 SNPs were synonymous and 12 were non-synonymous (Table 1). The dominant non-synonymous mutation Y133S reached or almost approached fixation in the three populations, with a frequency of 99.3, 100 and 95.1% in the China-Myanmar border, Myanmar and Thailand populations, respectively (Table 2). In addition, D637N was detected with high frequencies of 83.0, 36.5 and 68.3% in the China-Myanmar border, Myanmar and Thailand populations, respectively (Table 1). Other non-synonymous mutations were present in 0.74–7.84% of *pvhap2* sequences of the three populations (Table 1). Among the 12 synonymous mutations, A2636C (R849R) was present in 100% the parasites from the GMS (Table 1). Of note, no SNPs were observed in the putative functionally essential HAP2-GCS1 domain (codons 338–397 aa) of *pvhap2* gene (Table 1).

**Table 1:** Mutations and corresponding amino acid substitutions in the *pvhap2* gene

Nucleotide position	Codon	Wild-type	Mutant	Amino acid	Frequency (%)		
					China-Myanmar border (*n* = 135)	Myanmar (*n* = 51)	Thailand (*n* = 41)
77	26	GAT	GTT	D – V	0.74	0	0
78	26	GAT	GAA	D – E	0	0	2.44
183	61	GGG	GGC	**G – G**	0	3.92	0
479	129	AAG	AAC	K – N	37.04	7.84	0
487	133	TAC	TCC	Y – S	99.26	100	95.12
626	179	AGG	AGA	**R – R**	0	1.96	0
759	224	CAA	AAA	Q – K	1.48	0	0
797	236	GAC	GAT	**D – D**	0	1.96	0
1037	316	CAA	CAG	**Q – Q**	0	0	2.44
1038	317	GAG	AAG	E – K	0	0	2.44
1374	429	TTG	CTG	**L – L**	5.19	3.92	2.44
1624	513	TGC	TGT	**C – C**	0	1.96	0
1714	542	GGG	GAG	G – E	0	0	2.44
1808	573	TTA	TTG	**L – L**	0	1.96	0
1813	575	AAC	AGC	N – S	0	1.96	0
1998	637	GAT	AAT	D – N	82.96	36.54	68.29
2009	640	ACG	ACA	**T – T**	0.74	1.96	4.88
2139	684	GCC	GTC	A – V	0	1.96	0
2140	684	GCC	TCC	A – S	0	1.96	0
2226	713	GCC	ACC	A – T	0.74	1.96	0
2320	744	GGG	GTG	G – V	0	1.96	0
2357	759	ACG	ACA	**V – V**	0.74		2.44
2372	761	TCC	TCT	**S – S**	0	1.96	0
2384	765	AAA	AAG	**K – K**	0		2.44
2628	847	GGC	AGC	G – S	0	3.92	0
2636	849	CGA	CGC	**R – R**	100	100	100
2640	851	CCC	ACC	P – T	0	3.92	0
2649	854	GCA	ACA	A – T	1.48	1.96	0

*Notes*: The nucleotides and amino acid of synonymous mutation sites are indicated in bold

**Table 2:** Nucleotide diversity and summary statistics of *pvhap2* in the three GMS *P. vivax* populations

Locality	*S*	*η*	*NS*	*SP*	*k*	π ± SD	H	*Hd* ± SD
China-Myanmar border (*n* = 135)	10	10	7	3	0.589	0.00023 ± 0.00003	11	0.420 ± 0.052
Myanmar (*n* = 51)	17	17	9	8	1.363	0.00053 ± 0.00009	18	0.747 ± 0.057
Thailand (*n* = 41)	11	11	6	5	1.110	0.00043 ± 0.00007	9	0.705 ± 0.059
Total (*n* = 227)	28	28	16	12	0.924	0.00036 ± 0.00003	29	0.620 ± 0.032

*Abbreviations*: *S*, number of segregating sites; *η*, the total number of mutations; *NS*, number of non-synonymous polymorphisms; *SP*, number of synonymous polymorphisms; *k*, the average number of nucleotide differences; π, pairwise nucleotide diversity; H, number of haplotypes; *Hd*, haplotype diversity

*Note*: The extent of *pvhap2* gene sequence corresponded to nucleotides 13–2574 bp (codons 5–858, reference clone Sal I)

**Fig. 1:**
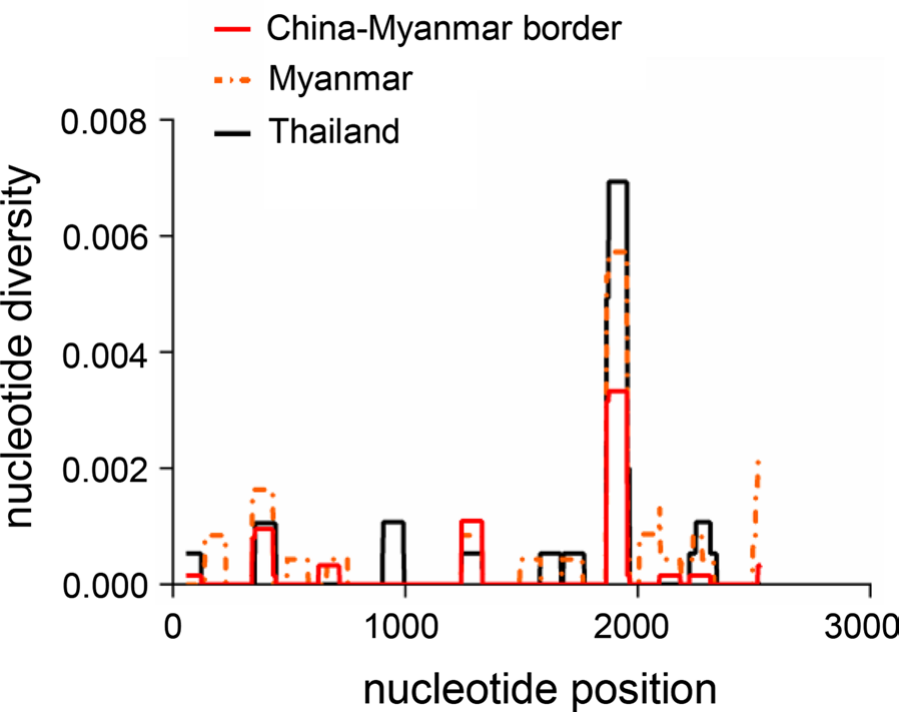
Nucleotide diversity across nucleotides 13–2574 region of *pvhap2* gene. The sliding window plot (90 bp window and 3 bp step size) of nucleotide diversity of *pvhap2* in China-Myanmar border, Myanmar and Thailand populations was used to achieve a high-resolution analysis. A total of 135, 51 and 41 sequences were used from China-Myanmar border, Myanmar and Thailand populations, respectively. Nucleotide positions are after the Sal I *pvhap2* reference sequence (PlasmoDB ID: PVX_094925)

The overall nucleotide diversity (π) for the 227 *pvhap2* sequences was low at 0.00036 ± 0.00003 (Table 2). The highest nucleotide diversity was observed in western Myanmar (0.00053 ± 0.00009), followed by Thailand (0.00043 ± 0.00007) and China-Myanmar border population (0.00023 ± 0.00003) (Table 2). Similarly, parasites from western Myanmar presented the highest haplotype diversity (Hd = 0.747 ± 0.057), whereas parasites from the China-Myanmar border showed the lowest Hd (0.420 ± 0.052) (Table 2). Sliding window plots of nucleotide diversity revealed π value distributed unevenly in *pvhap2* gene, with the highest π value detected in 1875–1953 bp region of the all three parasite populations (Fig. 1). Three (YNQD, SNQD and SNQN) out of five commonly observed global haplotypes of Pvhap2 according to the four amino acid replacements (Y133S, N575S, Q634P and D637N) were observed in these populations (Table 3). Of these, ‘SNQN’ was the most frequent haplotype in the China-Myanmar border (85.19%) and Thailand (68.29%) populations, while ‘SNQD’ was the common haplotype in Myanmar (62.75%) (Table 3). However, the Sal I type haplotype ‘YNQD’ was not observed in Myanmar, and was observed at very low frequencies in the China-Myanmar border (0.74%) and Thailand (2.44%) (Table 3).

**Table 3:** Distribution of Pvhap2 haplotypes among the three studied localities of GMS

Locality	Haplotypes Pvhap2		
	YNQD (Sal I)	SNQD	SNQN
China-Myanmar border (*n* = 135)	1 (0.74)	19 (14.07)	115 (85.19)
Myanmar (*n* = 51)	0 (0)	32 (62.75)	19 (37.25)
Thailand (*n* = 41)	1 (2.44)	12 (29.27)	28 (68.29)

*Notes*: The values represent the number and frequency (%) of found haplotypes on each studied locality. Haplotypes of Pvhap2 is according to four amino acid replacements on amino acid positions 133, 575, 634 and 637

When the *pvhap2* gene (nt 397–2097 bp) obtained from this study was aligned with previously published global *pvhap2* sequences [26], the highest average number of pairwise nucleotide (*k*) value was observed in the China isolates (1.267), followed by Thailand (0.963) and Myanmar (0.897) isolates, while the lowest *k*-value was detected in the PNG isolates (0.286) (Additional file 3: Table S2). Meanwhile, China and Thailand samples were found to have much higher nucleotide diversity and haplotype diversity (China, π = 0.00075 and Hd = 0.733 [26]; Thailand, π = 0.00057 and Hd = 0.668) than other global isolates (Additional file 3: Table S2).

### Evidence of natural selection

Several neutrality tests were performed to examine if the *pvhap2* gene was under selection. Except the China-Myanmar border isolates, which showed *d*_N_ = *d*_S_, negative values for *d*_N_-*d*_S_ were obtained for both Myanmar (− 0.0002) and Thailand (− 0.0003) populations; however, neither value was statistically significant by Fisher’s exact test (Table 5). Significant negative value of Tajima’s *D* (− 1.9897, *P* < 0.05) observed in Myanmar isolates indicated purifying or directional selection (Table 4). Although Tajima’s *D* value was also below zero in the China-Myanmar border and Thailand populations, the tests showed no significant departure from neutrality (Table 4). In comparison, both Fu & Li’s D* and F* tests identified significant deviation from zero in *pvhap2* genes in the three populations, suggesting expansion in population size and/or purifying selection (Table 4). Consistent results were obtained with the McDonald-Kreitman’s test using the *P. cynomolgi hap2* gene as the outgroup, which showed an excess of synonymous substitutions within the *pvhap2* sequences relative to synonymous substitutions from the outgroup species, albeit the result was significant only in China-Myanmar border population, suggesting functional constraint occurring at certain residues in this protein (Table 4).

**Table 4:** Summary statistics of *pvhap2* from the China-Myanmar border, Myanmar and Thailand *P. vivax* isolates

Locality	China-Myanmar border	Myanmar	Thailand
n	135	51	41
*d*_N_ ± SE	0.0002 ± 0.0001	0.0005 ± 0.0002	0.0004 ± 0.0002
*d*_S_ ± SE	0.0002 ± 0.0002	0.0007 ± 0.0002	0.0006 ± 0.0004
*d*_N_ - *d*_S_ (SE)	0.0000 (0.0002)	− 0.0002 (0.0003)	− 0.0003 (0.0004)
*P* (Z-stat)	0.0517	0.5133	0.6309
TD	− 1.6612	− 1.9897*	− 1.7165
D*	− 2.3305*	− 3.0110*	− 2.9330*
F*	− 2.4919*	− 3.1527*	− 2.9888*
NI (*P*)	4.746 (0.0349)	1.989 (0.2777)	2.441 (0.1916)

*Notes*: The variance of the difference between the numbers of synonymous (*d*_S_) and nonsynonymous (*d*_N_) substitutions per site was computed using the bootstrap method (500 replicates). Analyses were conducted using the Nei-Gojobori method in MEGA-X [27]

*Abbreviations*: TD, Tajima’s *D* test; D*, Fu & Li’s D* value; F*, Fu & Li’s F* value; NI, neutrality index value for the Mcdonald-Kreitman test; SE, standard error

*P < 0.05

The codon-based tests for selection were performed on *pvhap2* gene by using FEL [41], SLAC [41], and FUBAR [42] implemented in the Datamonkey webserver [32]. The likelihood-based algorithms (FUBAR method) revealed purifying selection at 640, 756 and 849 codons in the China-Myanmar border isolates (Table 5). Meanwhile, purifying selection was also detected by the FEL method in the Myanmar isolates at 61, 179, 246 and 513 codons (Table 5). The FUBAR method detected both positive and purifying selections in the Myanmar isolates at specific codons (positively selected sites: 129, 637 and 684; purifyingly selected sites: 61, 179, 236, 429, 513, 573, 640, 761 and 849) (Table 5). It is noteworthy that D637N was a prevalent mutation in the Myanmar population, reaching a frequency of 36.54% (Table 1). Whereas no positive selection was detected in the Thailand population with all the three methods, only purifying selection was detected at 765 and 849 codons by the FEL method, and at 316, 640, 756, 765 and 849 codons by the FUBAR method (Table 5).

**Table 5:** Codon-based tests for selection on *pvhap2* gene in three studied localities of GMS

Locality	FEL	SLAC	FUBAR
China-Myanmar border (n = 135)	Positively selected: none	Positively selected: none	Positively selected: none
	Negatively selected: none	Negatively selected: none	Negatively selected: 640, 756, 849
Myanmar (n = 51)	Positively selected: none	Positively selected: none	Positively selected: 129, 637, 684
	Negatively selected: 61, 179, 236, 513	Negatively selected: none	Negatively selected: 61, 179, 236, 429, 513, 573, 640, 761, 849
Thailand (n = 41)	Positively selected: none	Positively selected: none	Positively selected: none
	Negatively selected: 765, 849	Negatively selected: none	Negatively selected: 316, 640, 756, 765, 849

### Population differentiation

Pairwise comparisons were performed using the Wright’s fixation index (*F*_ST_) to study genetic differentiation between parasite populations. AMOVA revealed statistically significant differences in all studied populations. The pairwise comparison of parasite populations between the China-Myanmar border and Thailand populations, as well as between the Thailand and Myanmar populations showed little genetic differentiation with an *F*_ST_ value of 0.04077 and 0.09271, respectively (Table 6). Moderate genetic differentiation was observed between the China-Myanmar border and western Myanmar populations, and between Iran and Myanmar populations, with an *F*_ST_ value of 0.24833 and 0.19226, respectively (Table 6). In contrast, high-level genetic differentiation was detected between the China-Myanmar border and Iran populations (*F*_ST_ = 0.60266), as well as between Thailand and Iran populations (*F*_ST_ = 0.44161), suggesting a high degree of differentiation between Southeast and West Asia populations.

**Table 6:** Genetic differentiation (*F*_ST_) of the *pvhap2* among the four geographical populations

Locality	China-Myanmar border	Myanmar	Thailand	Iran
China-Myanmar border (*n* = 135)	–	0.00000	0.01488	0.00000
Myanmar (*n* = 51)	0.24833	–	0.00231	0.00000
Thailand (*n* = 41)	0.04077	0.09271	–	0.00000
Iran (*n* = 52)	0.60266	0.19226	0.44161	–

*Notes*: *F*_ST_ values are shown in the lower left quadrant and the *P*-values are shown in the upper right quadrant. *P* < 0.05 was considered as significant

### Haplotype network and phylogeny

**Fig. 2:**
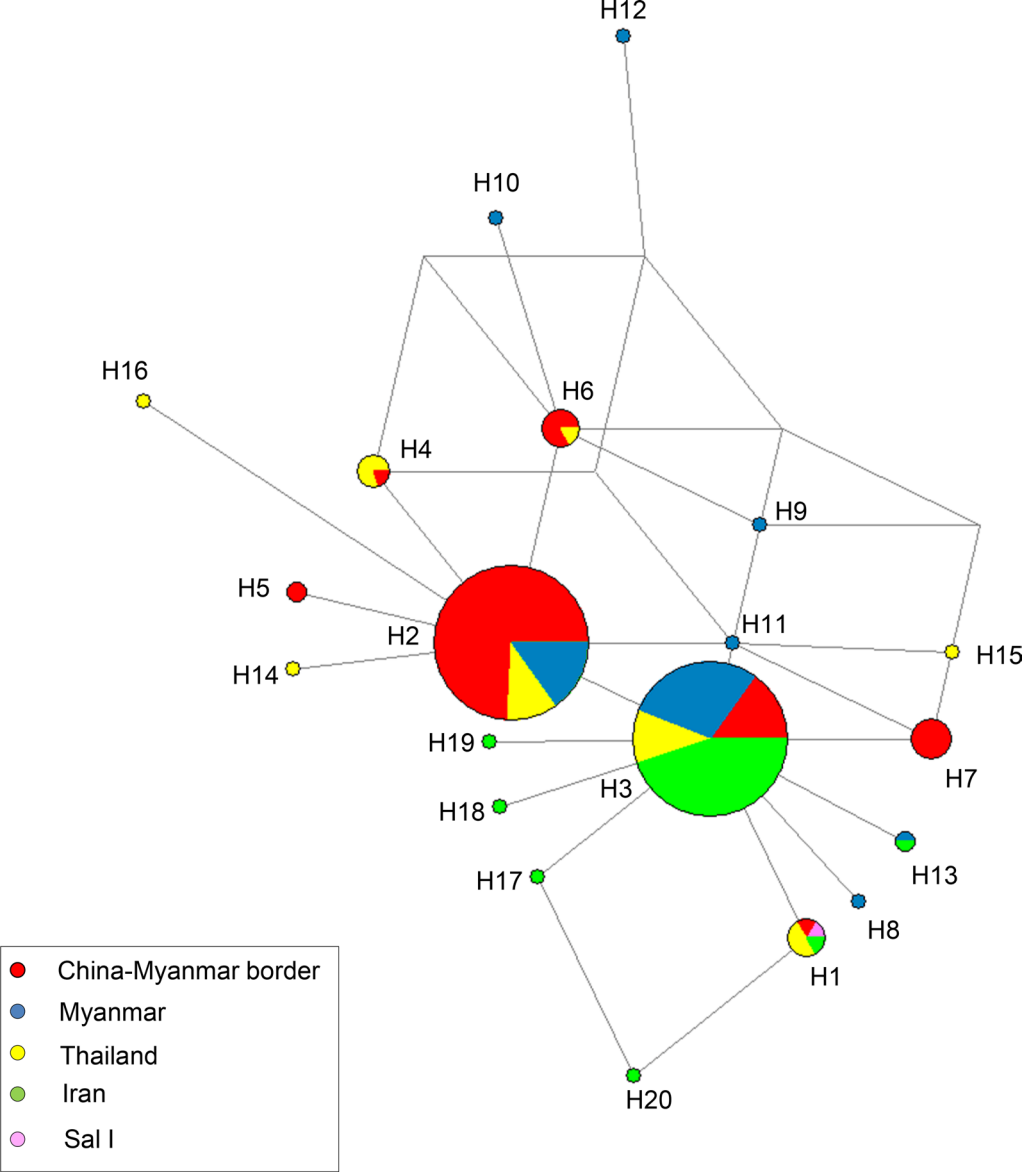
Haplotype network of China-Myanmar border, Myanmar, Thailand and Iran Pvhap2 sequences. China-Myanmar border (*n* = 135, red), Myanmar (*n* = 51, blue), Thailand (*n* = 41, yellow) and Iran (*n* = 52, green). The haplotype network was constructed using the median-joining method in Network 5.0.1.1. Totally, 20 haplotypes were detected in GMS *pvhap2* DNA sequences. The size of the circles is related to the haplotype frequency and the color indicates different countries. Lines separating haplotypes represents mutational steps

Haplotype network analysis showed that among 20 haplotypes of 279 *pvhap2* sequences obtained from the China-Myanmar border, western Myanmar, Thailand and Iran isolates, 60% (12/20) was represented by single parasite isolates (Fig. 2). Two major haplotypes H2 and H3 were most abundant, with a frequency of 50 and 35.7%, respectively (Fig. 2). H2 was shared among the three GMS parasite populations and reached a frequency of 77.0% in the China-Myanmar border population. H3 was shared among the three GMS and the Iran parasite populations and reached 88.2% (45/51) in the Iran parasite population. The prevalence of other haplotypes ranged from 0.4 to 2.5%. Parasites with the Sal I reference haplotype (H1) had a 2.1% prevalence, which was present in the China-Myanmar border, Thailand and Iran parasite populations with a frequency of 0.7%, 7.3% and 1.9%, respectively (Fig. 2).

**Fig. 3:**
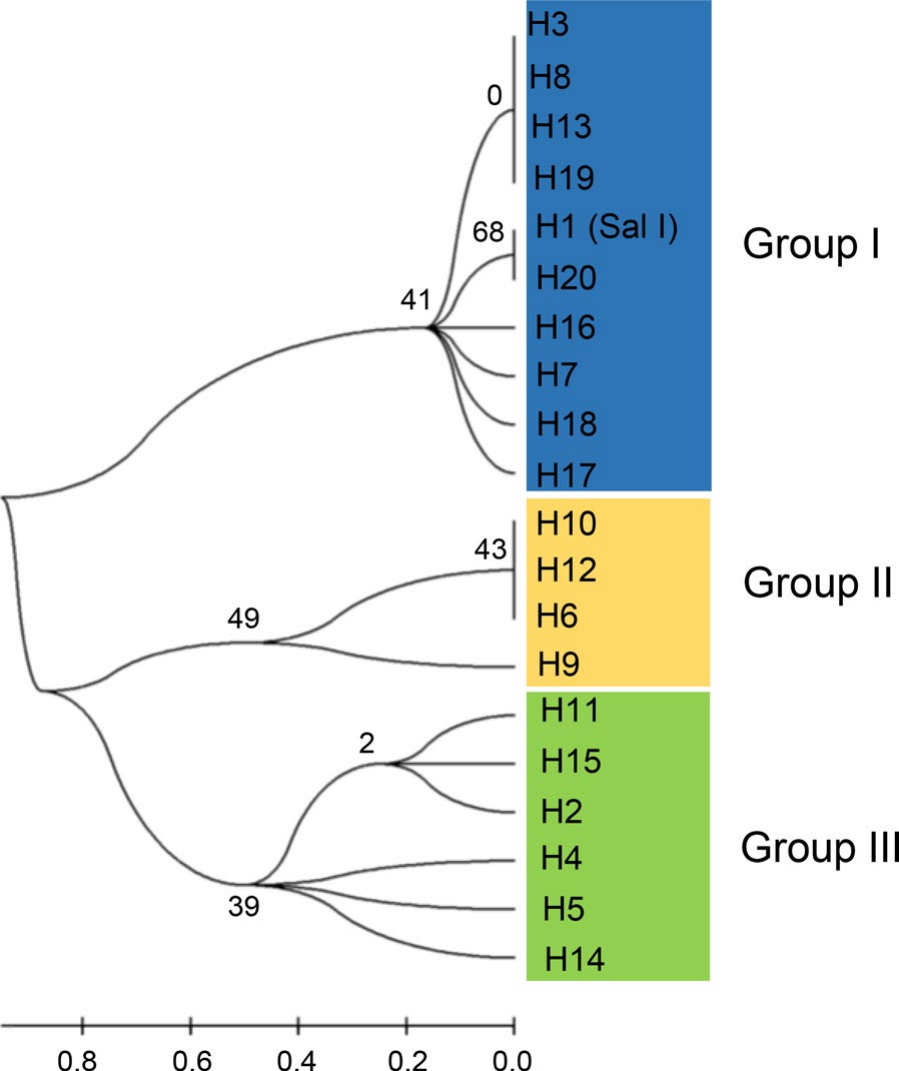
Phylogenetic analysis of Pvhap2 haplotypes from China-Myanmar border, Myanmar, Thailand and Iran isolates. Maximum likelihood tree for *pvhap2* sequences (310–2007 bp) in China-Myanmar border, Myanmar and Thailand isolates. The maximum likelihood tree was reconstructed based on alignment by ClustalW with bootstrap analyses to assess clade support (500 replicates) using a single consensus sequence for Pvhap2 for 20 haplotypes obtained from China-Myanmar border, Myanmar, Thailand and Iran isolates. The 20 haplotypes were clustered into three main groups, which are shown in blue (Group I), yellow (Group II), and green color (Group III), respectively

Phylogenetic analysis revealed that the haplotypes were clustered into three main groups with Group I consisting of 10 haplotypes, Group II consisting of 4 haplotypes, and Group III consisting of 6 haplotypes (Fig. 3). It is interesting to note that the neighbor-joining consensus tree for *pvhap2* revealed geographical clustering by countries or regions, with the Iran haplotypes only observed in Group I, whereas 75% of Group II haplotypes was unique to the western Myanmar population, and Group III consisted of admixed haplotypes from the three GMS populations (Fig. 3).

### Polymorphisms associated with B and T cell epitopes

**Fig. 4:**
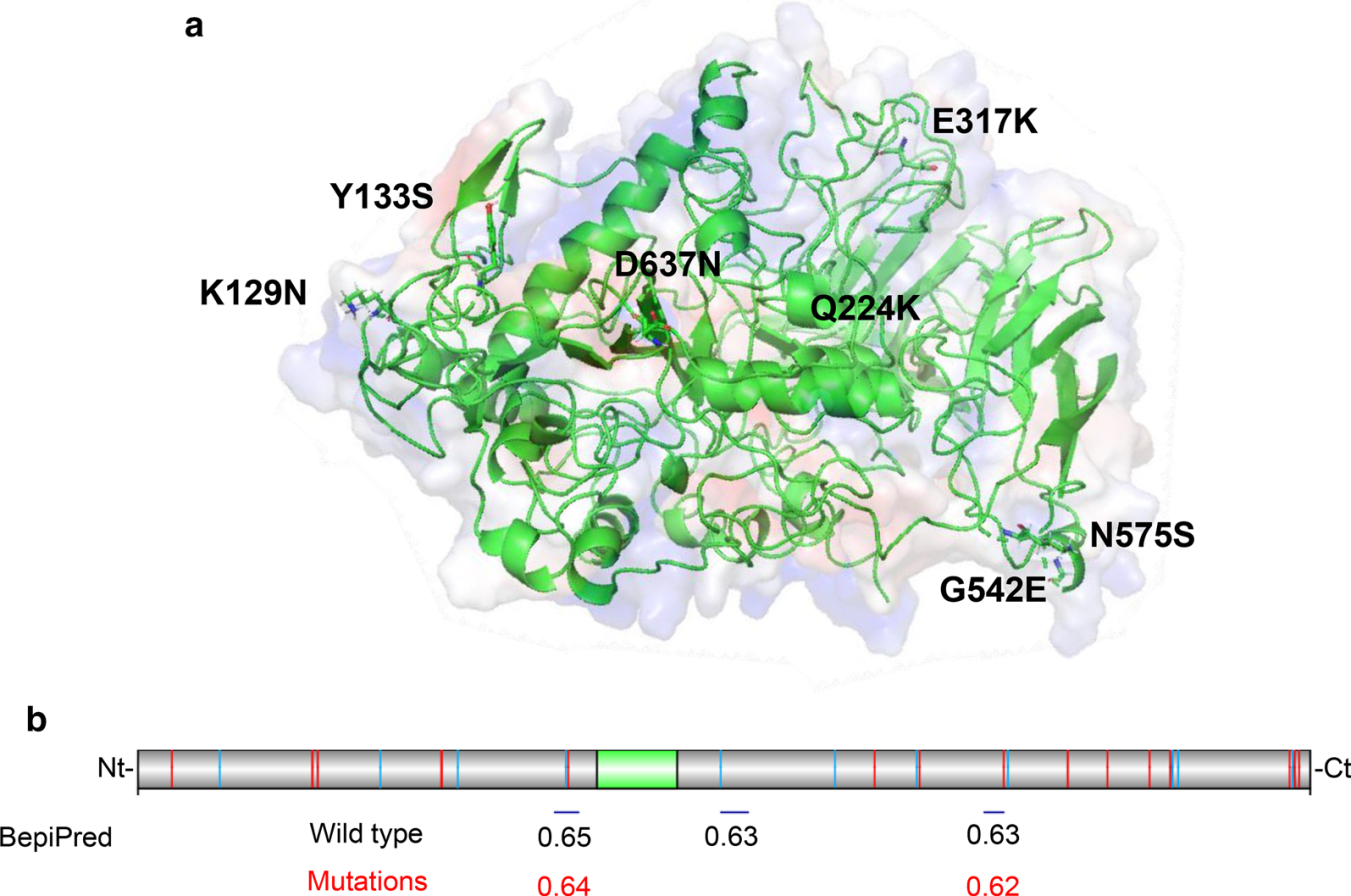
Modeling structure and epitope prediction analysis of Pvhap2. **a** Non-synonymous mutations within commonly analyzed PvHAP2 regions (129–637 aa) of global isolates are illustrated by text in black on the 3D structure of Pvhap2. The pinkish and blue clouds represent the negative and positive surfaces, respectively. **b** Primary structure of the Pvhap2 protein. DII that contain the ‘cd loop’ region is shown in green. Synonymous and non-synonymous mutations found in China-Myanmar border, Myanmar and Thailand are illustrated by blue bars and red bars, respectively. The blue lines represent predicted linear B cell epitopes. The N- and C-terminal amino acids (aa), and aa position of each B cell epitope were also indicated. The BepiPred values represent the predicted score of linear B cell epitope in the dominant haplotype (H2) of China-Myanmar border, Myanmar and Thailand (black numbers), and mutant strain (red numbers)

The secondary structure of deduced amino acid sequence of Pvhap2 was analyzed by using the Pymol software. Most residues, including K129, Y133, Q224, E317, G542, N575 and D637 are predicted to be exposed to the surface, except Q224 which would be hidden inside the negative pocket (Fig. 4a). All the mutation sites belonged to flexible loop structures (Fig. 4a). Three overlapped peptides (S307-G324, L429-P453, V623-D637) were predicted to be B epitopes from the BepiPred and ABCpred software. The prediction scores for the three epitopes (S307-G324, L429-P453 and V623-D637) were 0.65, 0.63, and 0.63, respectively. Two non-synonymous mutations were found in these predicted B cell linear epitopes (E317K and D637N), which resulted in slight decreases of the predicted scores for the epitopes (Fig. 4b).

## Discussion

Sequence analysis of the *pvhap2* gene revealed that the Y133S mutation at a high frequency in the parasite populations studied here was fixed in India, Thailand, Vietnam, China, North Korea and PNG, but was observed at lower frequencies in Colombia (26.1%), Peru (14.3%) and Mexico (46.6%) [26]. The G1998A SNP coding for D637N was predominant in isolates from the China-Myanmar border (82.96%), Thailand (68.29%), Thailand-2014 (42.48%), Vietnam (100%), China (50%), North Korea (100%), but had a lower frequency in western Myanmar (36.54%) and was absent in isolates from Iran, India, PNG, Mauritania-I, Brazil, Colombia, Peru and Mexico [26]. Previous reports have demonstrated the ‘cd loop’ region of HAP2 protein is essential for gamete fertilization [16]. Of note, two non-synonymous mutations (K129N and Y133S) were found within the loop region (108–222 aa) of the Pvhap2 protein, which corresponds to the HAP2 orthologues in *P. falciparum* (178–195 aa) and *P. berghei* (174–191 aa). Only one G626A SNP (synonymous mutation, R179R) was found within the hydrophobic “fusion loop” (cd loop) segment (167–198 aa) of loop region in Pvhap2. Given the essential function of the “cd loop” in mediating membrane fusion, these results suggest that functional conservation of the same region within the HAP2 protein in *P. vivax* may limit the accumulation of amino acid mutations [16, 22]. Whether these mutations influence Pvhap2 function or immunogenicity needs further investigations.

The divergent prevalence of Pvhap2 mutations in different endemic areas probably reflects the different demographic histories of the parasites such as endemicity and selection imposed by the human host and mosquito vectors. In the GMS, the Y133S mutation was fixed or almost fixed, whereas the D637N mutation showed regional differences in prevalence. Difference in regional malaria epidemiology may be held accountable for this divergence in D637N prevalence. Pairwise comparison showed considerable differentiation between China-Myanmar border and western Myanmar populations. Alternatively, this divergence may reflect an evolutionary trend of the parasite populations through time, as the parasites from western Thailand (83.0%), China-Myanmar border (68.3%) and western Myanmar (36.5%) were collected in 2011, 2016 and 2018, respectively. It is possible that this longitudinal decrease in D637N prevalence may be associated with host immunity since HAP2 is shown to be naturally immunogenic and antibodies against this protein are present in endemic populations [24]. Interestingly, codon-based selection tests detected positive selection at three codons in the western Myanmar parasites, including codon 637, suggesting potential targeting of these codons by host immunity. Of note, D637N is located in an epitope region (625–651 aa) of the Pvhap2 protein; thus, this mutation may alter the immunogenicity of the HAP2 protein. Future experiments are needed to show whether this amino acid substitution could interfere with fusion function (gamete fusion) or protein recognition by host antibodies.

*Pvhap2* showed lower genetic diversity (ranging from 0.00018 to 0.00075 for all global isolates analyzed) [26] compared to other sexual stage transmission-blocking antigens reported in *P. vivax* such as *pvs28/25* (π = 0.0034/0.0013, Yunnan Province of China, [43]; π = 0.0041/0.0023, Myanmar, [44]), *pvs48/45* (π = 0.00173, global isolates, [45]), and *pvs230* (π = 0.00118, global isolates, [46]). This high level of conservation may be a consequence of the essential function of this protein in fertilization. Although not significant, the negative value for the *d*_N_-*d*_S_ in nucleotide sequences of *pvhap2* gene from the GMS suggests purifying selection may act on this gene. Allele-frequency-based tests further indicate that *pvhap2* was influenced by purifying selection. Further, when zoomed in specific regions of the *pvhap2* gene, codons under purifying selection were identified in all three GMS parasite populations. This is similar to the gametocyte/gamete-surface antigen *pvs230*, which appears to be under purifying selection [46]. Another gametocyte/gametes surface protein, *pvs48/45*, was found to be under positive selection [47]. Thus, in addition to functional constraints, Pvhap2 may avoid immune selection pressure from the human host, as it is predominantly expressed by microgametes found in mosquito midgut, not in humans. Regardless, the evolutionary conservation of pvhap2 may be beneficial for the design of a TBV.

Despite the low level of sequence diversity, the overall genetic differentiation among GMS and Iran populations in this study was remarkable, which was cross-verified from *F*_ST_ and phylogenetic analysis. Within the GMS, however, low *F*_ST_ values among all GMS populations studied here may suggest extensive gene flow among these populations, possibly mediated or enhanced by migratory human populations [48]. These results also highlight that Pvhap2-based TBV design need to factor in the geographical differentiation of pvhap2 among populations. For example, GMS parasite populations did show a highly prevalent haplotype, H2 (frequency within the GMS area: 61.67%), whereas the Sal I Pvhap2 haplotype was not observed in Myanmar (0/51), and circulates with low frequency in the China-Myanmar border (1/135) and Thailand (3/41) isolates. Although the two non-synonymous positions (E317K and D637N) that are located in the predicted B cell epitopes did not seem to change the protein configuration and B cell epitope prediction score, this awaits further experimental confirmation.

## Conclusions

The present study revealed very low level of genetic diversity and purifying selection in *pvhap2*, indicating the importance of this protein in parasite’s survival and transmission. Little genetic differentiation identified among the three GMS populations suggests extensive gene flow between these areas. Notably, this study revealed a dominant haplotype (H2) of *pvhap2* in GMS isolates. Additionally, the present study identified potential B-cell epitopes with relatively low diversity. These findings provide important information for designing an effective TBV based on Pvhap2.

## Supplementary information

**Additional file 1: Figure S1.** Geographical distribution of *P. vivax* populations contributing to this study. *Plasmodium vivax* isolates from three sampling sites, including China-Myanmar border, Myanmar and Thailand were analyzed in the present study.

**Additional file 2: Table S1.** List of the primers along with their nucleotide sequences used to assess the genetic diversity of the *pvhap2* gene.

**Additional file 3: Table S2.***Pvhap2* gene polymorphism by country.

## Data Availability

The datasets supporting the conclusions of this article are included within the article and its additional files. The nucleotide sequences were deposited in the GenBank database under the accession numbers MT087312-MT087446 (China-Myanmar border), MT087488-MT087538 (Myanmar) and MT087447-MT087487 (Thailand).

## Abbreviations

GMS: Greater Mekong Subregion
TBV: transmission blocking vaccine
GCS1: generative cell specific 1
gDNA: genomic DNA
Sal I: Salvador I
*S*: number of segregation sites
*η*: total number of mutations
π: nucleotide diversity
*k*: average number of nucleotide differences
H: number of haplotypes
Hd: haplotype diversity
*d*_N_: mean number of non-synonymous substitutions per non-synonymous site
*d*_S_: mean number of synonymous substitutions per synonymous site
AMOVA: analysis of molecular variance
*F*_ST_: fixation index
SNP: single nucleotide polymorphism
