# Supplementary figures and images for: Plasmodium vivax HAP2/GCS1 gene exhibits limited genetic diversity among parasite isolates from the Greater Mekong Subregion
Source: Parasit Vectors. 2020 Apr 7;13:175. doi: 10.1186/s13071-020-04050-0 (PMC7137254; doi:10.1186/s13071-020-04050-0)

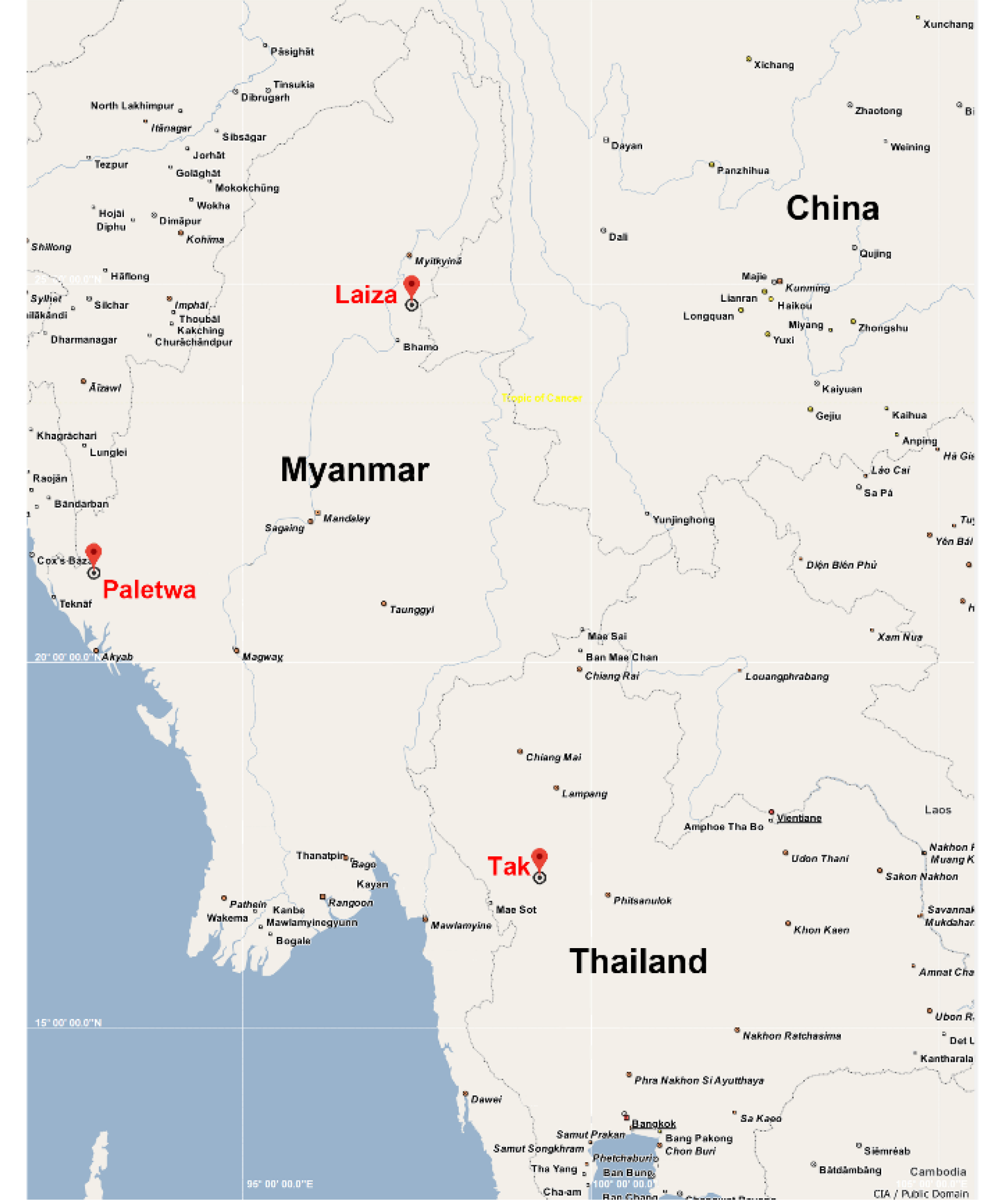

Supplement: Supplementary file 1 — Additional file 1: Figure S1. Geographical distribution of P. vivax populations contributing to this study. Plasmodium vivax isolates from three sampling sites, including China-Myanmar border, Myanmar and Thailand were analyzed in the present study. [file 13071_2020_4050_MOESM1_ESM.tif]
