# Supplementary material for: Plasmodium vivax HAP2/GCS1 gene exhibits limited genetic diversity among parasite isolates from the Greater Mekong Subregion
Source: Parasit Vectors. 2020 Apr 7;13:175. doi: 10.1186/s13071-020-04050-0 (PMC7137254; doi:10.1186/s13071-020-04050-0)
Supplement: Supplementary file 2 — Additional file 2: Table S1. List of the primers along with their nucleotide sequences used to assess the genetic diversity of the pvhap2 gene. [file 13071_2020_4050_MOESM2_ESM.docx]

**Additional file 2. Table S1** List of the primers along with their nucleotide sequences used to assess the genetic diversity of *pvhap2* gene.

| **Primers name** | **Usage** | **Sequence** |
| --- | --- | --- |
| pvsHap2 -1F | Primary PCR | 5′- GAGGCGTACATGCAGATAGA-3′ |
| pvsHap2 -1R |  | 5′- ACCCTGGTGGTGCGTAGAG -3′ |
| pvsHap2-nest-F | Nested PCR | 5’- ACGTAGGAGGAGAAGGAG-3’ |
| pvsHap2-nest-R |  | 5’- ATTCGCCTGGAGGTTCTA-3’ |
| pvsHap2-Fs1 | Sequencing | 5'- CATGTTCATGCCAATCGTAT-3' |
| pvsHap2-Rs1 |  | 5'- TAGTTACAAAGGAGAAGAAAT-3' |
| pvsHap2-Fs2 |  | 5'- CCTTCAGAGATATTACGATGC-3' |
| pvsHap2-Fs3 |  | 5'- GAAGGAGTGTTCCAAGG-3' |
| pvsHap2-Fs4 |  | 5'- TGGCAGAATGGGTAACAAG -3' |
| pvsHap2-Rs2 |  | 5'- TCGCGTGCCCTTATTAG -3' |
