# Supplementary material for: Plasmodium vivax HAP2/GCS1 gene exhibits limited genetic diversity among parasite isolates from the Greater Mekong Subregion
Source: Parasit Vectors. 2020 Apr 7;13:175. doi: 10.1186/s13071-020-04050-0 (PMC7137254; doi:10.1186/s13071-020-04050-0)
Supplement: Supplementary file 3 — Additional file 3: Table S2.Pvhap2 gene polymorphism by country. [file 13071_2020_4050_MOESM3_ESM.docx]

**Additional file 3.** **Table S2 *Pvhap2* gene polymorphism by countries.**

| Country (no.) | *S* | *η* | *k* | π ± SD | H | Hd ± SD | Reference |
| --- | --- | --- | --- | --- | --- | --- | --- |
| China-Myanmar border (135) | 6 | 6 | 0.515 | 0.00030 ± 0.00005 | 7 | 0.393 ± 0.051 | Current study |
| Myanmar (51) | 8 | 8 | 0.897 | 0.00053 ± 0.00011 | 9 | 0.599 ± 0.057 | Current study |
| Thailand (41) | 8 | 8 | 0.963 | 0.00057 ± 0.00009 | 7 | 0.668 ± 0.059 | Current study |
| Iran (52) | 5 | 5 | 0.302 | 0.00018 ± 0.00006 | 7 | 0.253 ± 0.080 | Current study |
| Thailand (Kanchanaburi, 14) | 4 | 4 | 0.956 | 0.00056 ± 0.00017 | 4 | 0.626 ± 0.110 | [[26](#_ENREF_26)] |
| China (6) | 3 | 3 | 1.267 | 0.00075 ± 0.00027 | 3 | 0.733 ± 0.155 | [[26](#_ENREF_26)] |
| Colombia (23) | 2 | 2 | 0.490 | 0.00029 ± 0.00008 | 3 | 0.423 ± 0.104 | [[26](#_ENREF_26)] |
| Peru (28) | 2 | 2 | 0.706 | 0.00042 ± 0.00006 | 3 | 0.611 ± 0.060 | [[26](#_ENREF_26)] |
| Mexico (15) | 1 | 1 | 0.533 | 0.00031 ± 0.00003 | 2 | 0.533 ± 0.052 | [[26](#_ENREF_26)] |
| PNG (7) | 1 | 1 | 0.286 | 0.00052 ± 0.00005 | 2 | 0.286 ± 0.196 | [[26](#_ENREF_26)] |

Number of the amino acid residues is according to Sal-I sequence. Comparison of genetic diversity was performed among nucleotide 397 – 2097 bp of *pvhap2* gene. The italic and shading letters indicates non-synonymous mutations. S: number of polymorphic sites (segregating sites), *η*: total no. of mutations, *k*: average number of pair-wise nucleotide differences, H: haplotypes based on nucleotides, Hd: Haploype diversity, π: observed average pairwise nucleotide diversity, SD: standard deviation.
